# How Does Information Processing Speed Relate to the Attentional Blink?

**DOI:** 10.1371/journal.pone.0033265

**Published:** 2012-03-26

**Authors:** Troy A. W. Visser, Jeneva L. Ohan

**Affiliations:** 1 School of Psychology, University of Queensland, Brisbane, Queensland, Australia; 2 School of Psychology and Counselling, Queensland University of Technology, Brisbane, Queensland, Australia; University of Sydney, Australia

## Abstract

**Background:**

When observers are asked to identify two targets in rapid sequence, they often suffer profound performance deficits for the second target, even when the spatial location of the targets is known. This attentional blink (AB) is usually attributed to the time required to process a previous target, implying that a link should exist between individual differences in information processing speed and the AB.

**Methodology/Principal Findings:**

The present work investigated this question by examining the relationship between a rapid automatized naming task typically used to assess information-processing speed and the magnitude of the AB. The results indicated that faster processing actually resulted in a greater AB, but only when targets were presented amongst high similarity distractors. When target-distractor similarity was minimal, processing speed was unrelated to the AB.

**Conclusions/Significance:**

Our findings indicate that information-processing speed is unrelated to target processing efficiency per se, but rather to individual differences in observers' ability to suppress distractors. This is consistent with evidence that individuals who are able to avoid distraction are more efficient at deploying temporal attention, but argues against a direct link between general processing speed and efficient information selection.

## Introduction

A chief requirement for effective visual perception is the ability to select relevant information from rapidly shifting sensory inputs. It is unsurprising then that decades of research have been devoted to understanding the mechanisms that accomplish this task. While much of this work has focused on understanding visual search for targets amongst distractors spread across space, a rapidly-growing literature has begun to examine this question in the temporal domain as well. In the majority of these studies, observers are presented with a temporal analogue of a visual search task known as rapid-sequential visual presentation (RSVP). Here, observers are presented with a stream of sequential distractors, within which are embedded one or more target items. This setup yields good accuracy for an initial target (T1); however, identification of a second target (T2) is generally impaired when it follows T1 closely in time (<∼500 ms). This second target deficit is known as the attentional blink (AB).

From a theoretical standpoint, the AB deficit is informative because it directly polls the temporal dynamics of conscious vision, and reveals the relatively slow time course over which awareness emerges [1]. Practically, AB performance has also been linked to a number of domains including reading [2], video gaming [3], and psychiatric disorders such as schizophrenia and anxiety [4], [5]. This suggests that the AB may be tapping key mechanisms underlying a variety of both normal and abnormal behaviors. A key question, however, is how the AB, and temporal processing more broadly, are linked to individual differences in general cognitive abilities.

Many researchers have argued that the AB deficit reflects variations in general information processing speed. For example, Hari and Renvall suggested that the link between reading impairments and the AB was rooted in slower target processing (increased “dwell time of attention”) that negatively impacts both reading fluency and the AB [6]. Similarly, capacity-limited models of the deficit, such as the two-stage model [7] tie the second target deficit to the time required to process the first target. This implies that individuals who are generally quicker target processors should have an advantage over their slower processing peers.

Some evidence in favor of this point comes from studies that have shown a relationship between T1 task difficulty or T1 processing speed and the magnitude of the AB [7]–[12]. However, a relationship between difficulty and the AB has not been obtained in all studies [13]–[15]. Moreover, some researchers have argued that the relationship between difficulty and the AB reflects task switching ability rather than target processing speed per se [16]. Beyond these uncertainties, it must also be noted that even if the AB does depend on target processing speed, this does not necessarily imply that it would depend on the speed of information processing more generally.

Potentially more informative are studies that have focused on individual differences in the AB. Here, results suggest a subset of individuals, referred to as “non-blinkers”, reliably escape the deficit [17] as a result of superior skills at ignoring irrelevant information presented during the RSVP stream (e.g., [17]–[20]). In turn, this skill has been tied to event-related potential (ERP) evidence that non-blinkers consolidate target information in short-term memory at a faster rate [21]. While this work again suggests a link between target processing speed and the AB, it is still unclear whether this advantage reflects faster information processing more generally. This caveat is further highlighted by the fact the performance advantage for non-blinkers seems to disappear when faced with targets that are not alphanumeric stimuli [17] or that are presented in the auditory domain [22].

Finally, a number of studies have directly tackled the question of how information processing speed and the AB are linked by examining correlations between AB magnitude and independent measures of processing speed. As in the work reviewed above, however, results have been equivocal. For example, in samples drawn from university populations, Gillard-Crewther, Lawson, Bello & Crewther [23] found that faster processing speed led to a greater AB, while Arnell, Howe, Joanisse, & Klein [24] found no relationship between these factors. In a combined sample of participants with normal reading and dyslexia, Badcock, Hogben and Fletcher [25] found slower processing speed led to a greater AB. However, this relationship was not significant when either dyslexics or normal readers were considered in isolation. Finally, Rizzo, Akutsu and Dawson [26] also found slower processing speed led to a greater AB in a combined sample of normal participants and participants with focal cerebral lesions. However, this effect was only marginally significant, and again disappeared when normal participants or lesion participants were considered in isolation.

In considering the uncertain picture presented by the studies discussed above, it is important to note that the vast majority of these experiments used RSVP paradigms with relatively high target-distractor similarity. As a result, it is impossible to determine whether processing speed plays a role in the AB in the absence of confusable distractors. This is a particularly critical question because a robust AB can be obtained when distractors are omitted [27] or when they share minimal target similarity [28]. Moreover, theories that relate processing speed and the AB deficit do not posit that this relationship is dependent on the presence of confusable distractors. Therefore, in the present work we focused on determining how processing speed was related to the AB both in the presence and absence of confusable distractors.

To address these questions, we assessed AB performance across trials with both low and high target-distractor similarity, and measured information processing speed using a rapid automatized naming (RAN) task similar to those employed by Badcock et al. [25] and Arnell et al. [24]. In RAN tasks, observers are asked to verbally identify a series of visual stimuli (e.g., letters, color patches) as quickly and accurately as possible. Performance on these tasks has been linked to speed of information processing [29]–[31], and in the case of the letter identification variant used here, speed of lexical access in particular [32]. We used a letter-naming RAN task, rather than other types of stimuli, because observers were also asked to identify letters during the AB task.

To assess the degree of relationship between the AB deficit and processing speed, we initially correlated RAN performance with two indices of AB performance – a measure of the impact of target-distractor similarity on the AB, and a measure of AB magnitude. We then confirmed the nature of these correlational findings by comparing AB performance across the range of inter-target intervals (lags) between groups comprising the top and bottom halves of the distribution of RAN scores.

## Methods

### Participants

Participants were recruited via a first-year introductory psychology research pool or word-of-mouth (N = 69; 59.4% female). Ages ranged from 16 to 27 years (M = 20.72, SD = 3.36). First-year participants received course credit to compensate them for their one-hour participation. All participants reported normal or corrected-to-normal (i.e. glasses or contact lenses) vision and had English as a first language.

### Ethics Statement

All experimental procedures were conducted in accordance with the principles expressed in the Declaration of Helsinki, and were approved by the University of Queensland Ethics Committee. Written informed consent was obtained from each participant prior to participation.

### Apparatus & Stimuli

To assess RAN performance, we used the rapid letter-naming task from the Comprehensive Test of Phonological Processing (CTOPP) [32]. Participants were asked to read aloud a series of randomly arranged letters (A, T, S, K, C and N) presented on two A4 cards, in a 9×4 arrangement, as quickly as possible. The total time taken to read all of the letters was recorded. For the AB task, stimuli were presented on an Acer AC915 monitor running at a refresh rate of 100 Hz, slaved to a Pentium computer running Presentation software (Version 12.4, Neurobehavioral Systems). Targets were letters (except I, O, Q and Z due to their similarity to digits). Distractors were random-dot patches (Low-Similarity condition) or digits (High-Similarity condition). There were 10 different random-dot patterns, and different digits (1–9). Target masks consisted of the keyboard symbols @, #, and %. These masks followed targets in both the Low- and High-Similarity conditions in order to avoid confounding different levels of target-distractor similarity with effectiveness of target masking. All items were approximately 1° square, light grey in color, and presented on a black background.

### Procedure

Participants completed the AB and RAN tasks in counterbalanced order. The RAN task was completed in a brightly lit room. The AB task was completed in a dimly-lit room, with participants seated approximately 60 cm from the computer monitor.

**Figure 1:**
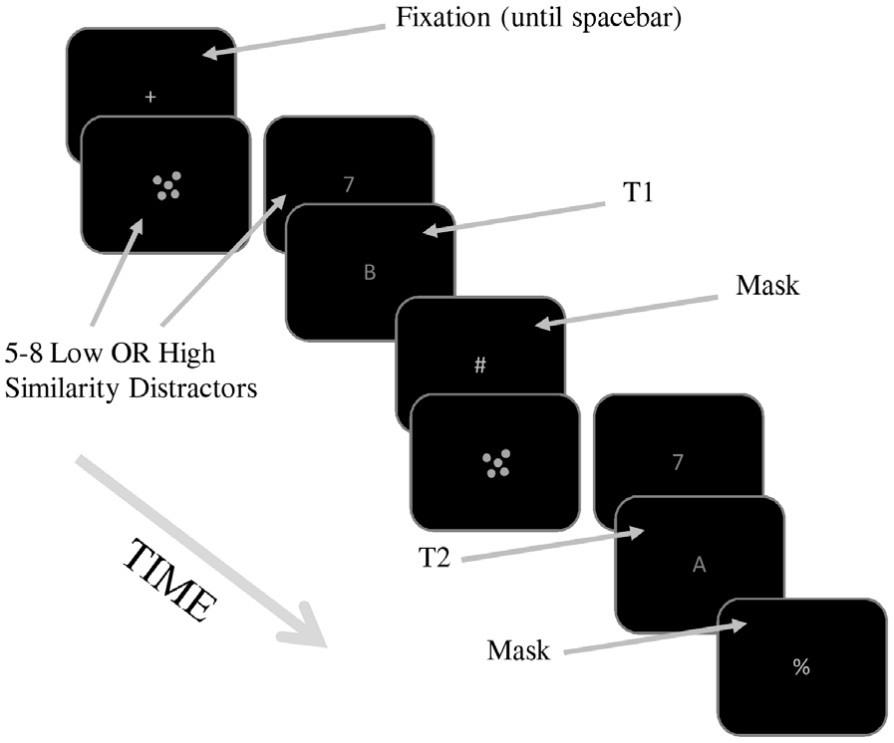
Schematic illustration of the sequence of events on a Lag 3 trial (not to scale). Participants were instructed to identity the two letters in the sequence when the last item disappeared.

The sequence of events on a typical trial is illustrated in Figure 1. Each trial began with a central fixation cross. Participants were then instructed to focus their gaze at fixation, and press the space bar to begin the trial. This initiated an RSVP stream at fixation. Each item in the stream was presented for 30 ms and followed by a blank screen for 70 ms, yielding a presentation rate of 10 Hz. After 5 to 8 distractors, T1 was presented, and then followed by: a) T2 (at lag 1); b) a mask, one distractor and T2 (lag 3); or, c) a mask, five distractors, and T2 (lag 7). A keyboard-symbol mask always followed the second target, which was the final item in the RSVP stream (thus, the total length of the RSVP stream varied with lag). After the offset of the mask, participants were prompted to identify the two target letters by typing them into the keyboard. Participants were told to enter the target letters in any order, and responses were scored without consideration of response order. Following these responses, the fixation cross re-appeared, indicating the next trial was ready to begin. Each participant completed 15 trials at each combination of lag and target-distractor similarity (randomly intermixed), resulting in a total of 90 trials.

## Results

Preliminary examination of the data indicated two participants had RAN scores that were over 2.5 standard deviations above the sample mean, while an additional two participants had an AB magnitude (see below) greater than 2.5 standard deviations above the sample mean. Data from these participants were omitted from all further analyses.

### Correlations

To examine the correlation between processing speed and AB performance, we first created an index of the impact of target-distractor similarity on the AB by subtracting mean T2 accuracy scores (conditionalized on T1 accuracy) on trials with high similarity from those with low similarity. We then calculated an index of AB magnitude, separately for trials with low- and high-similarity distractors. Our index was based on the methodology employed by Raymond, Shapiro and Arnell (1995). For each participant, we subtracted T2 accuracy (given T1 correct) scores from 100% at each lag. We then added the resulting difference scores to yield an estimate of overall AB magnitude.

**Figure 2:**
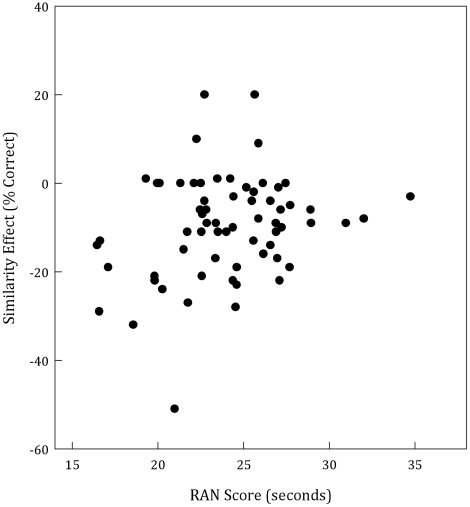
Scatter plot of Similarity Effect, calculated as the difference between T2 accuracy (given correct T1) in the low- and high-similarity conditions summed across lags, and mean RAN scores. Negative Similarity Effect scores indicate a larger AB when target-distractor similarity was high.

**Figure 3:**
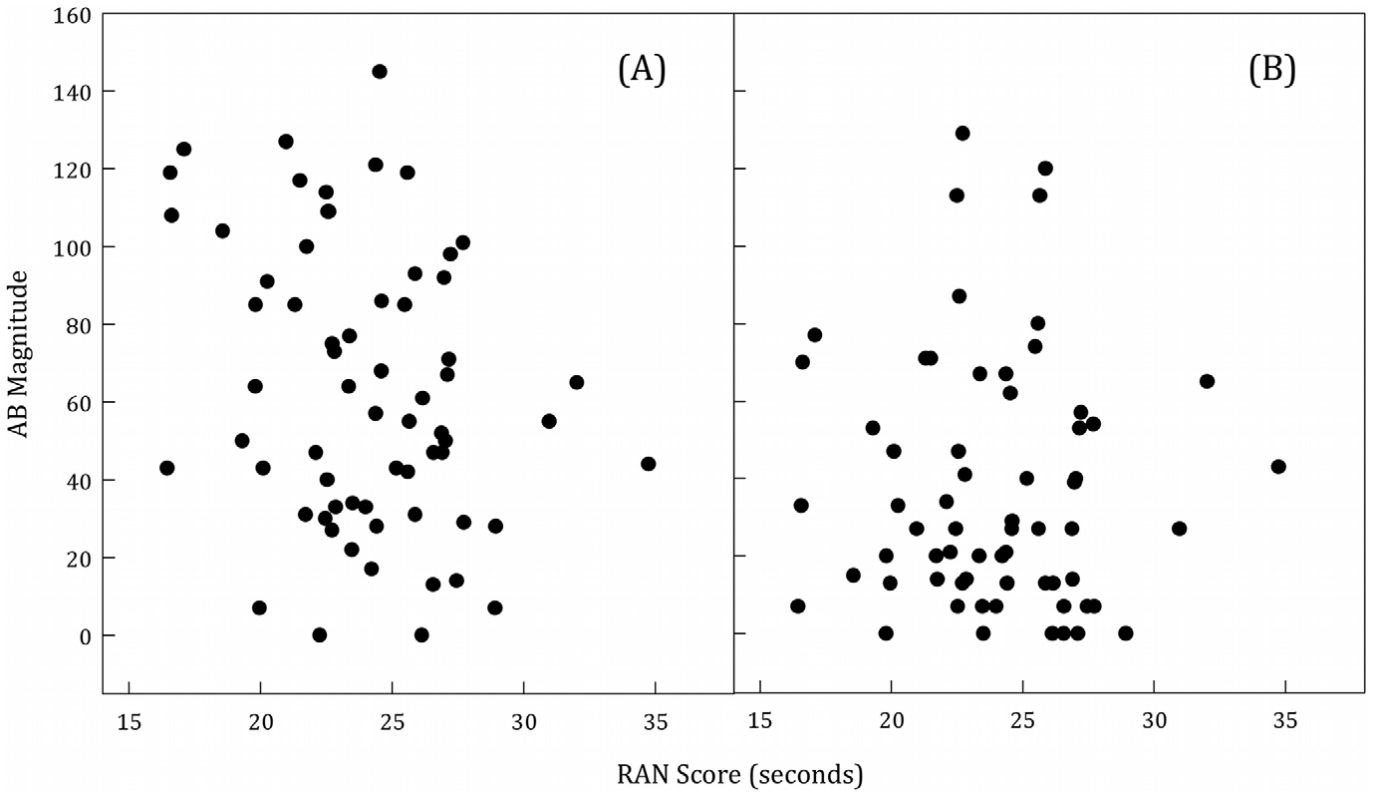
Scatter plots of AB Magnitude, calculated as the difference between 100% and T2 accuracy (given correct T1) summed across each lag, and mean RAN scores. The plot on the left (A), represents this relationship when target-distractor similarity was high. The plot on the right (B) represents this relationship when target-distractor similarity was low.

After calculating our indices, we then calculated partial correlations between these indices and RAN performance, controlling for participant age. This yielded a significant negative correlation between processing speed and the impact of target-distractor similarity, *r*(62) = −.272, *p* = .03, such that faster processing speeds were related to greater decrements in performance with high similarity distractors (see Figure 2). We then looked at the relationship between RAN performance and AB magnitude. In the high-similarity condition, faster processing speed was related to greater AB magnitude, *r*(62) = −.316, *p*<.02 (see Figure 3A). Critically, however, in the low-similarity condition, this relationship was non-significant, , *r*(62) = −.076, *p*>.54 (see Figure 3B). This difference suggests that in the absence of high-similarity distractors, information processing speed is unrelated to the AB. Note that we did not conduct correlational analyses for T1 because overall accuracy was near ceiling (90.27%), thus limiting performance range, and the size of possible correlations.

### Analyses of Variance (ANOVAs)

Although the AB indices here encapsulate the overall deficit shown by participants, it is useful to supplement correlational analyses by examining both T1 and T2 performance over lags as a function of RAN performance. To do this, we divided our participants into two groups based on a median split of RAN scores, and then compared target accuracy for the top and bottom performing groups as a function of lag and target-distractor similarity.

For T1, the resulting 2 (Group: Fast, Slow)×3 (Lag: 1, 3, 7)×2 (Similarity: High, Low) mixed-design ANOVA yielded main effects of Lag, *F*(2, 126) = 4.35, *p*<.02, *η^2^* = .07, and Similarity, *F*(1, 63) = 40.06, *p*<. 001, *η^2^* = .39, as well as an interaction between these factors, *F*(2, 126) = 8.87,*p*<.001, *η^2^* = .12. Examination of the data suggests that when similarity was high, T1 accuracy was lower at Lag 7 than earlier lags (Lag 1: 87.1%, Lag 3: 87.5%, Lag 7: 84.7%). However, this difference was significant only between Lags 3 and 7, *t*(64) = 2.38, *p* = .02. When similarity was low, accuracy increased across lags (Lag 1: 91.1%; Lag 3: 95.9%; Lag 7: 96.5%). These differences were significant between Lags 1 and 3, *t*(64) = 3.89, *p*<.001, and Lags 1 and 7, *t*(64) = 4.63, *p*<.001. No other main effects or interactions were significant (*p*>.46, *η^2^*<.02).

**Figure 4:**
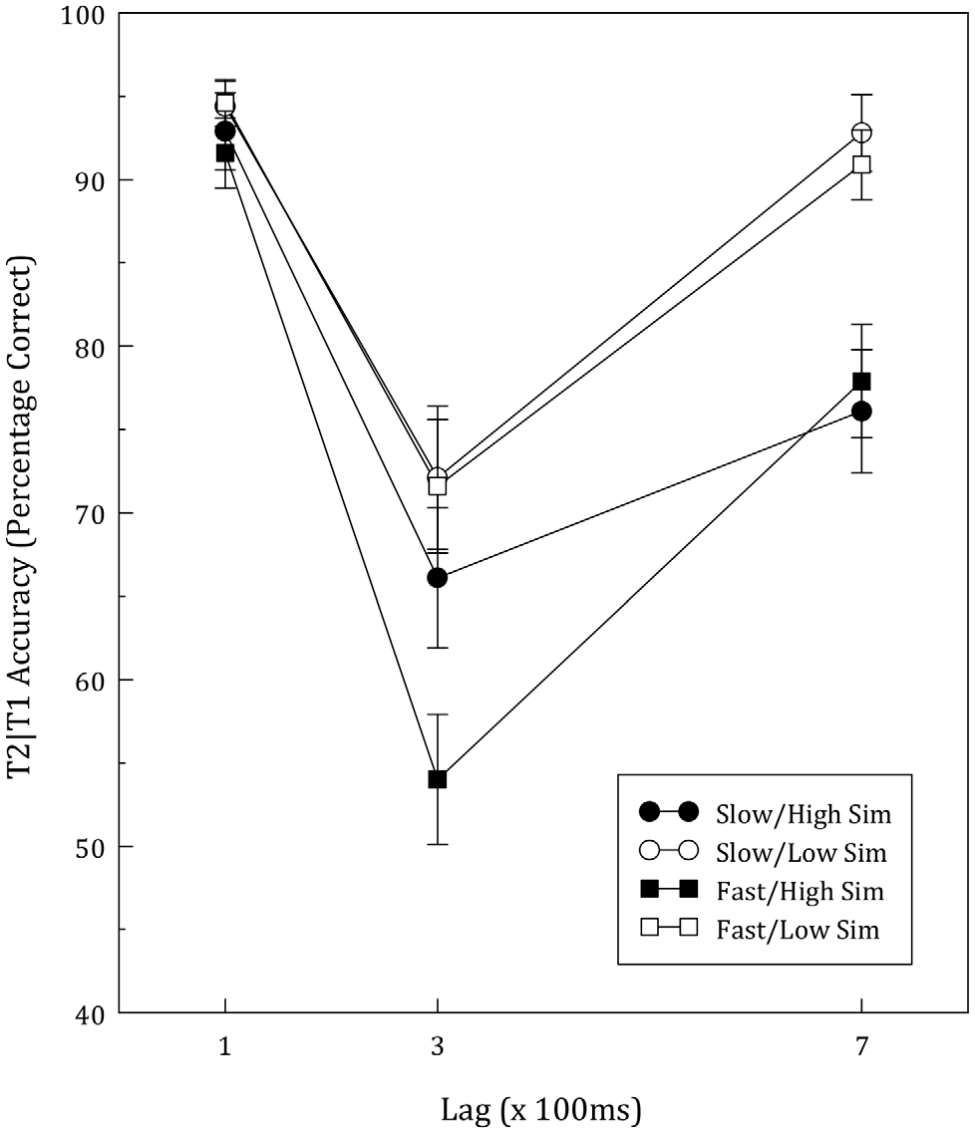
Second target accuracy (given correct T1) as a function of RAN performance, target-distractor similarity, and lag. “Fast” and “Slow” refer to groups comprising the top and bottom half of mean RAN scores. “High” and “Low” refer to level of target-distractor similarity (i.e., digit vs. random dot distractors). Error bars represent one standard error of the mean.

For T2 (conditionalized on T1 correct), the same ANOVA yielded main effects of Lag, *F*(2, 126) = 72.07, *p*<.001, *η^2^* = .53, and Similarity, *F*(1, 63) = 43.78, *p*<.001, *η^2^* = .41, as well as interactions between Lag and Similarity, *F*(2, 126) = 10.28, *p*<.001, *η^2^* = .14, and Lag, Similarity, and Group, *F*(2, 126) = 3.64, *p*<.03, *η^2^* = .06. No other main effects or interactions were significant (*p*>.29, *η^2^*<.02). On the basis of the three-way interaction obtained in this analysis and the different pattern of correlations found with similar and dissimilar distractors, we conducted separate analyses of low- and high-similarity distractor trials. As can be seen in Figure 4, on low-similarity trials, the Group×Lag mixed-design ANOVA revealed only a main effect of Lag, *F*(2, 126) = 50.01, *p*<.001, *η^2^* = .44. However, the same analysis for high-similarity trials confirmed earlier correlational results, showing a significant main effect of Lag, *F*(2, 126) = 58.11, *p*<.001, *η^2^* = .48, and an interaction between Group and Lag, *F*(2, 126) = 3.03, *p* = .052, *η^2^* = .05, which included a significant quadratic component, *F*(1, 63) = 5.21, *p*<.03, *η^2^* = .08, indicative of the deeper deficit at Lag 3 experienced by those with lower RAN scores. These analyses show that participants who process information more quickly have a much more prominent AB, but only when target-distractor similarity is high.

## Discussion

The purpose of the present study was to investigate the relationship between information processing speed and temporal attention. Such a relationship is often assumed by AB researchers and consistent with several theoretical accounts [7], [9]. However, existing studies in this area have yielded decidedly mixed results, and have not directly addressed whether AB performance is related to processing speed in the absence of high target-distractor similarity. To address this issue, the present study assessed information processing speed using a letter variant of the widely-used RAN task and related this to indices of the impact of distractor similarity on target accuracy and AB magnitude.

The basic findings were consistent with previous AB studies. Accuracy for both targets was impaired when target-distractor similarity was high [28], [33]. Performance at Lag 1 was also reliably better than at Lag 3. Such “lag-1 sparing” was also expected in the absence of attentional switches between targets [34]. Most importantly, however, we found that faster processing speed was related to increased AB magnitude, but only when high-similarity distractors were included in the RSVP. When similarity was low, this relationship disappeared. These results indicate that the core AB deficit – that is the failure to attend to the second target as a result of first-target processing – is not reflective of a general slowing in information processing.

At first glance, the fact that processing speed is unrelated to the AB in the absence of high-similarity distractors may seem surprising given various studies that have directly linked T1 processing time with AB magnitude [8], [11]–[12]. However, such a relationship would only be expected if target-processing time were primarily determined by information processing speed alone. In fact, the total time required to process a target seems to vary distinctly as a function of many factors including working memory load [35]–[36], stimulus contrast [12], and task-switching [37]. Thus, as discussed earlier, it is in fact likely that T1 processing time is not a reasonable proxy for information processing speed more generally.

The evidence here for dissociation between target processing time in the AB and general processing speed also argues for an important caveat in the interpretation of AB studies. Namely, as mentioned earlier, researchers have often assumed that an increase in AB magnitude or depth is symptomatic of a more general reduction in information processing speed [2], [6]. However, while such a relationship is both sensible and possible, it is not necessarily true. In fact, as discussed below, the results here suggest that a more reliable index of a slow-down in information processing would be observing increased costs of target-distractor similarity on AB magnitude between different experimental groups.

The fact that RAN performance was strongly related to the AB only when target-distractor similarity was high suggests a relationship between information processing speed and temporal attention that is profoundly linked to distraction, rather than to direct modulation of target selection. This idea is broadly consistent with earlier evidence that the ability to inhibit distractors is beneficial for AB performance [18]–[20]. It is also interesting that we found that greater information processing speed actually led to poorer performance in the presence of high similarity distractors. This relationship is broadly similar to what was found by Gillard-Crewther et al. [23] in an AB task with high target-distractor similarity. However, the results are the opposite of the relationship proposed by Martens et al. [21] and to results obtained by Badcock et al. [25] and Rizzo et al. [26]. Moreover, they are also apparently inconsistent with the null findings reported by Arnell et al. [24].

Why might these inconsistencies have arisen? With respect to the results of Badcock et al. [25] and Rizzo et al. [26], it is possible that differences are driven by the fact that our sample comprised normal participants, while a large proportion of their participants came from abnormal populations. This suggestion is supported by existing evidence for dissimilarities between normal and dyslexic participants related to the AB. Namely, while the AB reliably interacts with reading ability in a normal sample [38], this interaction is absent in dyslexic samples [25], [39].

With respect to the results of Arnell and colleagues [24], closer examination of their paradigm, in which participants viewed RSVP streams of numbers, letters, pictures and colour patches, suggests that target-distractor similarity may have been substantially less than in the high-similarity condition reported here. First, the pool of distractors used by Arnell et al. [24] was about half the size of the one used here. This opens up the possibility that distractors could be more effectively recognized and filtered by participants [40]. It is also notable that performance on the 25% of trials with colour targets was substantially higher than in the other conditions. This is consistent with an explanation in terms of reduced target-distractor similarity. Taken together then, it is reasonable to conjecture that overall target-distractor in Arnell et al. [24] was more similar to our low-similarity condition than to our high-similarity condition. In turn, then, the absence of a significant correlation between processing speed and AB magnitude is perfectly in keeping with the findings reported here.

Finally, with respect to Martens et al. [21], one possible explanation for our dissimilar findings is that their observers were only presented with high-similarity RSVP streams. Thus, although non-blinkers in this task showed an earlier P3 peak, it is impossible to ascertain whether this was actually related to distractor processing, since the nature of distractors was not manipulated. It may be that the earlier P3 peak was instead related to some other characteristic of non-blinkers that was beneficial to their AB performance. Another possible explanation for the differing outcomes is that the non-blinkers in Martens et al.'s study [21] were a highly select sample of experienced observers in RSVP tasks, comprising the top 8% of performers from an original sample of 207 individuals. Given evidence for practice effects in RSVP paradigms [41], [42], these expert observers may have approached the task or processed information differently than our broader based sample of naive undergraduates.

Of course, it still remains to explain why faster information processing speeds might lead to a more significant AB. One explanation, advanced by Gillard-Crewther et al. [23], is that slower processors may encode information more durably, thus partially avoiding limitations in working memory thought to underlie the AB. While sensible, however, this would not explain why this relationship was only obtained when target-distractor similarity was low.

As an alternative, we suggest that it may be useful to consider a number of different lines of previous research related specifically to distraction. First, Visser et al. [28] argued that distraction has negative consequences in the AB task because highly-similar distractors pass an “input filter” designed to speed target selection (this is similar to the idea of attentional control settings in the spatial capture literature) [43], [44]. As a result, these distractors occupy resources meant for the target, and delay target processing in a manner akin to actual targets in a conventional AB paradigm. Also relevant is the work of Lavie and colleagues [45], [46] who argued that distraction occurs when observers have too many attentional resources available and thus automatically attend to irrelevant material, and Olivers and Nieuwenhuis [47] who suggested that in difficult RSVP tasks, observers “over-commit” attentional resources on the RSVP stream, and thus process distractors as well as targets, leading to poorer target performance.

Putting these elements together, it would seem that a reasonable explanation for the negative relationship between information processing speed and AB performance is that fast information processors have too many resources available to devote to incoming RSVP items. As a result, they not only process targets, but also distractors in the manner outlined by Visser et al. [28]. Distractor processing, in turn, leads to poorer target performance for both T1 and T2. Such an explanation is consistent with a specific effect of processing speed only in the presence of high-similarity distractors. It is also broadly consistent with earlier arguments about the importance of inhibiting distractors in successful AB performance. One way to test this proposal directly would be to repeat the present study but to distract participants with concurrent music [47] or an irrelevant visual display presented concurrently with the RSVP stream [48]. This should reduce participants' ability to over-invest in the RSVP task, and thus ameliorate the relationship between information processing speed and the AB in the presence of high-similarity distractors.

In conclusion, the present study has shown that the AB deficit is unrelated to at least one measure of general information processing speed. Rather information processing speed affects the AB indirectly by modulating the likelihood that highly-similar distractors will capture attention. These results not only provide additional insights into the question of how temporal attention is related to general cognitive abilities, they also place important boundary conditions on how to relate performance on the AB task to general abilities. In future work, it would be beneficial to replicate this work with additional measures of information processing speed in order to establish the boundary conditions governing the relationship between speed and attention. It is also necessary to test the framework advanced here to explain the negative impact of processing speed on the AB. That said, our findings further highlight the importance of considering individual differences in explaining cognitive and perceptual phenomena, and we hope that they will spur new inquiries in this direction.
